# COVID testing hesitancy among pregnant patients: Lessons learned from the COVID-19 pandemic about the unique needs and challenges of medically complex populations

**DOI:** 10.21203/rs.3.rs-3892181/v1

**Published:** 2024-02-01

**Authors:** Ruth Farrell, Caitlin Dahler, Rachel Pope, Ellen Divoky, Christina Collart

## Abstract

Background Pregnant patients were a significant population to consider during the pandemic, given the impact of SARS-CoV-2 infection on obstetric outcomes. While COVID testing was a central pillar of infection control, it became apparent that a subset of the population declined to test. At the same time, data emerged about pregnant persons also declining to test. Yet, it was unknown why pregnant patients declined tests and if those reasons were similar or different from those of the general population. We conducted this study to explore pregnant patients' attitudes, access, and utilization of COVID-19 testing to support healthcare for infection prevention management for this unique and medically complex population. Methods We conducted a qualitative study of patients who were currently or recently pregnant during the early stages of the pandemic and received outpatient prenatal care at one of the participating study sites. An interview guide was used to conduct in-depth telephone interviews. Coding was performed using NVivo, and analysis was conducted using Grounded Theory. Results The average age of the participants (N = 37) was 32 (SD 4.21) years. Most were < 35 years of age (57%) and self-described as White (68%). Qualitative analysis identified themes related to barriers to COVID-19 testing access and use during pregnancy, including concerns about test accuracy, exposure to COVID-19 in testing facilities, isolation and separation during labor and delivery, and diminished healthcare quality and patient experience. Conclusions The implementation of widespread and universal COVID testing policies did not address the unique needs and challenges of pregnant patients as a medically complex population. It is important to understand the reasons and implications for pregnant patients who declined COVID testing during the current pandemic to inform strategies to prevent infection spread in future public health emergencies.

### Background

Pregnant patients were a significant population to consider during the pandemic. On the one hand, they represented a medically complex population who was initially excluded from studies to understand how to prevent and manage COVID [1, 2]. On the other, data quickly emerged describing that pregnant patients were at increased risk of hospitalization from COVID-19 and, when hospitalized, increased chance of admission to the ICU and intubation [3–9]. Infants born to pregnant persons infected by SARS-CoV-2 also experienced adverse outcomes [10]. Thus, there was an emergent need to develop effective public health strategies to prevent and manage COVID-19 among this patient population.

SARS-CoV-2 testing was important strategy to control viral spread during the COVID-19 pandemic [11]. Despite strategies for widespread testing, studies reported barriers to implementing effective testing protocols across different patient groups and communities, including data about individuals who refused COVID testing [12–14]. These studies raised key questions about the reasons behind test refusal and the role of trust and inequity in the acceptability and use of COVID testing [15–18]. At the same time, reports emerged of pregnant patients who also declined COVID testing [19, 20]. However, it was unclear whether barriers to COVID testing among this population were similar to or different from those of non-pregnant individuals and communities. Additionally, there was also a need to identify the ramifications of test refusal for pregnant patients, particularly among those populations who faced poor obstetric outcomes and high infant mortality rates [21, 22].

Given the far-reaching medical, ethical, and social implications of COVID-19 for pregnant patients, children, families, and communities, it is important to understand pregnant patients’ attitudes and experiences with COVID-19 testing and to identify barriers for patients to obtain COVID-19 testing. We conducted a study to explore pregnant patients' perspectives, access, and utilization of COVID-19 testing during the pandemic to establish individual and system-level improvements to support healthcare for infection prevention management for the duration of the pandemic and future public health emergencies of similar magnitude.

## Methods

All research procedures were approved by the Cleveland Clinic Healthcare System Institutional Review Board under a Reliant Review Process. We recruited pregnant patients at outpatient centers within the Cleveland Clinic and University Hospital Healthcare System between March and September 2021. Inclusion criteria included patients who were 18 years of age or older, English speaking, had a viable intrauterine pregnancy, could provide informed consent for study participation, and received prenatal care at a participating healthcare facility. Participants were contacted using a recruitment letter sent to eligible participants via U.S. postal mail or the patient contact function of the electronic medical record (e.g., MyChart). Recruitment continued until thematic saturation was reached.

After an informed consent process, each participant participated in a telephone interview to maintain consistency with the healthcare systems' recommendations for social distancing and patient contact for research purposes at the onset of the pandemic. Interviews were conducted by two members of the research team using a semi-structured interview guide. The interview guide contained questions about patients' perceptions of COVID-19 testing during the pandemic. It was developed by content experts in obstetrics, infectious disease, public health, and ethics. With the participants' permission, the interviews were audio recorded and transcribed verbatim for analysis.

Qualitative analysis was approached as an iterative and progressive process of data immersion, coding, memoing, and theme identification consistent with Grounded Theory [23, 24]. We identified content domains and categories in transcripts to create a coding tree and used it to organize the data. A companion codebook was created to serve as a reference for the analysis. The coding and analysis processes were led by two study team members (R.F. and C.D.) using NVivo (version 12; QSR International). The research team held weekly meetings to review data coding and memoing and identify themes.

## Results

Thematic saturation was achieved with 37 interviews. The average age of the participants was 32 (SD 4.21) years. Most participants were < 35 years of age (57%), self-described White (68%), and from the same geographic area (Table 1). Qualitative analysis identified the following primary themes: (1) the fear of isolation and separation, (2) concerns about diminished patient experience and healthcare quality, (3) fear of viral exposure during the testing process, (4) a sense of guilt and stigmatization from a positive COVID test result, and (5) uncertainty and actionability of COVID test results. Illustrative quotes are listed below, with additional data presented in Table 2.

Table 1
Demographics

| Demographics of participants | Total (N = 37), n (%) |
|---|---|
| Mean age (years) | 32.03 ± 4.21 |
| Non-AMA | 21 (57) |
| AMA | 16 (43) |
| Race | |
| White | 25 (68) |
| Black | 5 (13) |
| Multiracial | 4 (11) |
| Asian | 2 (5) |
| Missing | 1 (3) |
| Hispanic or Latina | |
| No | 33 (89) |
| Yes | 3 (8) |
| Missing | 1 (3) |
| N of children | |
| 0 | 17 (46) |
| 1 | 16 (43) |
| 2 | 2 (5) |
| 3 | 1 (3) |
| 4 | 1 (3) |
| 5+ | 0 (0) |
| AMA: advanced maternal age | |

Table 2
Themes and example quotes

| Theme | Participant Quote |
|---|---|
| Fear of isolation and separation | *“I think if policies were policies that made sense to people, and there was no fear of saying like … for me… right like my fear was that my husband won’t [sic] be able to be with me when I had my first baby and that was exactly what happened.”* (ID-19)<br><br>*“Tell them they're not going to take your babies away. I mean, that's the fear. I mean, imagine if you especially like me and first-time mom and you took my baby way after I just had her?”* (ID-24)<br><br>*“I was in a [social media] group for my pregnancy for people due around the same time as me. I’d seen a lot of people only declining because they didn’t want it to affect their … at least to have one person to be able to be at the hospital with them. And a lot of people thought that, if you tested positive, your baby was taken from you.”* (ID-01)<br><br>*“I was scared at the results. I looked online and I saw some hospitals recommended you quarantine away from your baby for 14 days if you’re positive and then I had seen other things online that said that wasn’t recommended. Because of the bond being so in my head I was going through all the scenarios like, ‘U oh. What if I am positive? What would I choose? Would I quarantine away from my baby or, you know what I mean, like how would I prevent them from getting it from me?’ So yeah, a lot of thoughts went through my head in case I was positive.”* (ID-05) |
| Diminished patient experience and healthcare quality | *“If, you know, they feel that if their result is positive then they may be treated differently and not have access to care that they would otherwise get if they were unknown or negative.”* (ID-15)<br><br>*“I think just like general distrust of the medical system and being worried that it [test result] may be used against them in some fashion.”* (ID-21)<br><br>*“I couldn’t ever get fast service either. They had to completely gown up and put all of the masks and gear on before they could come see me. So, it was rough.”* (ID-04)<br><br>*“They didn't have any answer for my questions. They made them up on the spot because I was the first person in [de-identified hospital] to test positive during delivery.”* (ID-19) |
| Fear of viral exposure during the testing process | *“I do like that they separate those that think that they actually have it versus those that are getting tested because of a procedure.”* (ID-01)<br><br>*“I don’t know because I feel like even if you did have a testing center that was strictly for pregnant women, it still would potentially, you know even if they didn't have it, there could be other pregnant women there with it that would expose them in the same way. You know what I mean?”* (ID-12)<br><br>*“I think that they should definitely make a separate office for pregnant women to get them tested right then and there in office. Because going into that line and waiting and stuff when there’s still that fear because […] there is still not enough testing to know if it affects children or not during pregnancy. I think just for that reassurance, if they're feeling the symptoms, to test them right then and there that day.”* (ID-20)<br><br>*“When I went to get the test, I felt like it was very close to the other people who were getting tested. At the time, I thought I was probably negative and so I was worried I was going to get COVID by going to the testing site.”* (ID-21) |

| Theme | Participant Quote |
|---|---|
| A sense of guilt and stigmatization from a COVID positive result | *"We didn't know much about it [COVID], but I was the one who kind of brought it along closer to my family. So, I was nervous, I was scared, I was feeling guilty, you know? I had a lot of emotions going on when I found out."* (ID-16)<br><br>*"I was the first person to get it around like my friends, my family, I was the first person to get it. So, you know, I kind of also felt like the, the outbreak, I guess. I was the only one that had already had it, everyone was talking about it. We didn't know much about it, but I was the one who kind of brought it along closer to my family. So, I was nervous. I was scared. I was feeling guilty, you know. I had a lot of emotions going on when I found out."* (ID-23)<br><br>*"I think just the that guilt they don't want [...]. If they are positive, they don't want the guilt of what can...will the 'XXX' harm the baby?"* (ID-23) |
| Uncertainty and actionability of COVID test results | *"I wonder how much those women really knew about what was going on and what the risks were. You know, were they sticking their head in the sand, and like trying to ignore this situation, or were they just not educated enough?"* (ID-35)<br><br>*"When they told me my results were positive, I was really, really distraught. I started crying and, like, almost yelling because I was terrified. You know, the thing I've heard about the most is people dying from COVID. You know, it'd be deadly. Then I'm pregnant on top of that and I hear that, you know, women of color are higher risk for dying [...]. I was just freaking out altogether. I was horrified."* (ID-25)<br><br>*"They're scared. They really don't want it to come back positive. So, it's better not to know then to know."* (ID-15)<br><br>*"When I was asked about the causes and what can harm the baby there was no information for me really because that [the pandemic] had just started."* (ID-23) |

## Fear of Isolation and Separation

Study participants expressed their reasoning, hesitancies, and opinions toward the consequences of knowing a positive test result. The fear of separation and isolation was the primary driver in the decision to decline testing. Many participants spoke of the fear that, if they had a positive test result, they would be separated from their partner or key support person during labor. There was also the concern that, given the time-sensitive nature of the issue, they would not be able to find a replacement support person if their designated support person was not allowed in the delivery room. As described by this participant, *"I was very nervous to test positive going into pregnancy because then I know that it would like lockdown, you know, your husband can't come with you. You deliver kinda by yourself*" (ID-01*)*. Some participants reflected on their experiences and the experiences of other patients who shared stories such as this on social media: "*My fear was that my husband won't be able to be with me when I had my first baby, and that was exactly what happened. They made him go get tested, and he also tested positive. He also had COVID the same time as me [...]. The prenatal clinic was like, 'Now you [referring to her husband] tested positive. You're out'"* (ID-19).

Another significant concern was the fear that they would not be able to spend the first few moments or days of life with their newborn. While none of the participants experienced this personally, they spoke from collective fears and stories from other patients. *"A lot of people thought that if you tested positive*

*your baby was taken from you"* (ID-06). As such, this concern motivated some participants to avoid testing altogether or, in some cases, proceed with testing under significant stress. As this participant suggested to alleviate concerns about isolation from the newborn, *"Tell them [pregnant patients] they're not going to take your babies away. I mean, that's the fear. I mean, imagine if you, especially like me and a first-time mom, and you took my baby way after I just had her"* (ID-24).

### Concerns about Diminished Patient Experience and Healthcare Quality

Participants expressed different opinions about the experience of being treated as a COVID-positive patient during labor and delivery. Many of these concerns were due to personal protective equipment (PPE) requirements. For example, participants were concerned about delays in care that may result from providers needing to don PPE to prevent COVID-19 spread from a positive patient to healthcare providers. This raised the concern of potential delays in the provider being prepared for delivery, particularly in an emergent situation. As described by this participant, *"I didn't want to have to worry about if I needed them and hit the button waiting two minutes for them to try to hurry up and gown up to the whole PPE requirement for COVID. I wanted, if I hit a button, for them to be able to come into the room and take care of me*" (ID-24). Participants noted the potential fast pace of events during labor and delivery and the sense that their providers would attend to them immediately despite the uncertainties of the pandemic. As described by this participant, *"Because you're giving birth, there's no delays. There are already unknowns going into the birthing to begin with. I didn't want to wait for people to have to hurry up and gown up before they could come into my room. I wanted them to just come in with the normal stuff they were required to wear to come in*" (ID-19).

The need for additional PPE impacted the patient experience for some, particularly at the onset of the pandemic when donning procedures were being implemented: "*It was scary [...]. I think I was the only one on the floor with coronavirus when I delivered so that … and they had even told me they had maybe 15 pregnant women that had tested positive at that point. So, there wasn't a lot of knowledge about it [...]. I couldn't ever get fast service either. They had to completely gown up and put all of the masks and gear on before they could come see me. So, it was rough"* (ID-09).

### Concerns about Being Identified as COVID-19 Positive: Guilt and Stigmatization from a COVID Positive Result

Participants spoke of their concerns about the consequences of being diagnosed with COVID-19 during pregnancy and how those fears could influence the decision to undergo testing. This concern was due, in part, to the emotions they could have experienced if they learned that they had the virus and its effects on the fetus. For this patient, a positive COVID-19 diagnosis would introduce a series of stressors and questions about the decisions taken in the proceeding weeks: *"What did I do wrong? I've been careful. I think I took on a lot of that guilt on myself"* (ID-35).

Participants discussed the possibility of stigmatization from family and community members if they learned of the COVID-19 positive result. This included concerns about being blamed for choices that may have led to viral exposure and a sense that *"everyone was looking at you"* (ID-17) for having COVID-19 during pregnancy. There was also concern that the ramifications of a positive result may present a barrier to healthcare. This participant described why patients may decline testing: *"They feel that, if their result is positive, then they may be treated differently and not have access to care that they would otherwise get if they were unknown or negative"* (ID-21). As captured by this participant who had a COVID during pregnancy, *"It just was a little bit uncomfortable…like [COVID-positive diagnosis] made you feel different, like you were being treated differently"* (ID-10). In response to their experience, they recommended, *"Maybe not try to make people feel like they're being treated like they have a disease or something. And then be a little more understanding and sympathetic to the person who's sick"* (ID-10).

## Fear of Viral Exposure at Testing Facilities

Another central theme identified was the testing process and how accessibility, uncertainty, and exposure were of concern. Some participants feared that they would be exposed to the virus while presenting to a testing facility for asymptomatic COVID-19 testing before admission to Labor and Delivery, where other people getting tested were symptomatic: *"When I went to get the test I felt like it was very close to the other people who were getting tested […] so I was worried I was going to get COVID by going to the testing site" (ID-15).* The fear of exposure was particularly relevant to pregnant patients who were encouraged to socially isolate and convert in-person obstetric care to telehealth visits as primary ways to prevent exposure. This concern prompted participants to discuss the need for separate testing facilities for those who needed testing clearance for pregnant patients preparing for delivery. As described by this participant, *"I think that they should definitely make a separate office for pregnant women to get them tested right then and there in office because going into that line and waiting and stuff when there's still that fear…I think just for that reassurance if they're feeling the symptoms to test them right then and there that day" (ID-14).* Participants also spoke of their concerns about how to get tested while also caring for their children. As described by this participant who was scheduled for asymptomatic testing before a procedure, *"I don't want to bring my two-year-old in where a bunch of people are going to get tested"* (ID-01).

## Uncertainty about the Actionability of COVID Test Results

Additionally, participants discussed their doubts about the accuracy of COVID testing. Participants discussed the possibility of a false positive or false negative result. "*Am I positive or am I negative?' So, I think they just want to avoid the whole situation altogether"* (ID-16). Those concerns also included the fear of being told they were COVID-positive because of their symptoms and then the test coming back negative. As described by this participant, *"I think just the false negatives, you know. Women going to get tested and then they're come back positive and then the next day they're coming back negative."* (ID-16). These concerns added a new layer of worries about pregnancy and how to maintain their health during the pandemic.

Their worries about test accuracy continued as self-testing became available. While this new option gave patients more flexibility in the testing process, it also led some participants to question the use of self-administered versus healthcare-administered tests. As described by this participant, *“It made me nervous that I wasn't going to do it right. What I didn't realize is that I had to self-administer. So, it was going the drive through. They give you the test. You have to swab yourself. They have to watch you do it. The gentleman was like, "You know, you're not doing it right. You got to get it up a little farther.' So, I think that was my main concern is, ‘Oh goodness, like I'm not going to do this right.’ But nope, it ended up coming back and being positive. Should I even be [...] giving myself my own test?"* (ID-22).

This scenario left patients in another dilemma of feeling safe at home where outside exposures were eliminated, but a new fear of completing the test accurately themselves arose. Participants called for more education and information to support trust in the testing process and consistency with testing procedures. As described by this participant, "*So I guess my only concern is why the tests are different [...]. I feel like it should just be one protocol throughout the whole state"* (ID-17).

They were also concerned about the actionability of testing, specifically what evidence-based steps could be implemented if they were diagnosed with SARS-CoV-2. The possibility of a positive result in the context of numerous unknowns about COVID-19 was another factor that influenced whether patients would pursue testing or not. Along with that concern was the *"fear of the unknown"* (ID-13) and lack of consistent information about what patients would have to do if they tested positive for COVID-19, as stated by this participant: *"In my head, I was going through all the scenarios like, 'Uh oh. What if I am positive? What would I choose? Would I quarantine away from my baby or how would I prevent them from getting it from me? So yeah, a lot of thoughts went through my head in case I was positive"* (ID-03). This sense of uncertainty in the setting of a COVID positive result was described by this participant, *"Maybe they're declining because there was nothing that could be done, and they were maybe afraid to find out if they were. There's nothing that truly can be done for them [pregnant patients with COVID] or their baby. So, why put yourself under some kind of stress that you're ... you know, it's out of your hands"* (ID-04).

## Discussion

The COVID-19 pandemic brought to light several questions about how to develop and operationalize an effective method to identify patients with a novel virus and its impact on pregnant patients. This included how to anticipate and address obstacles to COVID testing, as issues of trust and transparency with healthcare and public health systems rapidly emerged with the onset of the pandemic, particularly among underserved and minority populations [25]. Many concerns centered on the interplay between public health ethics and medical ethics, when the need to address infection control at a population level departed from what many patients were accustomed to considering when encountering the medical system before that time [26].

While questions of individual choice and autonomy were paramount for all patients and communities during the pandemic, they were particularly salient for pregnant patients. This special population could not defer healthcare during the pandemic, requiring consideration for infection control for patients and healthcare providers when presenting for in-person outpatient and inpatient medical services. It was also a population for whom mandatory COVID testing was enacted at several healthcare centers, something not robustly applied in the non-gravid population. At the same time, pregnant patients were subject to delays in evidence-based infection prevention and management strategies because of initial exclusions of pregnant persons from studies during the early stages of the pandemic [2]. Despite these unique aspects, there was little data to understand how trust and transparency issues would manifest in this population and the impact of those factors on pregnant patients' ability and willingness to undergo COVID testing. Such data are essential to implementing and maintaining infection control strategies during the different waves of the COVID-19 pandemic and planning for future public health emergencies.

Our findings demonstrated that pregnant patients in our study shared many of the same concerns as non-gravid patients and communities. Reasons cited included: doubts about test reliability, the implications of a positive result, and the ability to make voluntary decisions about accepting or declining testing [13]. However, repercussions and stressors associated with those concerns were nuanced for pregnant individuals. For instance, the risk of SARS-CoV-2 exposure when presenting to a testing center was a reason observed for non-gravid individuals who refused testing. This concern was also reported among the pregnant participants in this study. They discussed this concern in the context of policies enacted about COVID testing specific to pregnant persons. These policies recommended universal testing for pregnant patients, including asymptomatic patients, before admission for delivery [17, 27]. In addition, many healthcare systems enacted a policy of treating pregnant patients who declined testing as COVID positive to prevent viral spread among healthcare staff. The fear of being exposed in the testing process and spreading the virus among their family (including children and elderly adult relatives) compared with the fear of being treated as if COVID positive while, in fact, COVID negative, presented a decisional dilemma for participants and raised important questions for them about the integrity of their autonomy during pregnancy.

In addition, pregnant patients in this study reported similar concerns as non-gravid individuals about test accuracy, the ramifications of a positive result, or the chance that the result may be a false positive. One of the primary concerns about a positive result was the implications of isolation from others to prevent infection spread. Fears of isolation and quarantine were documented across populations during the pandemic [28–30]. Yet, there was scant data on what isolation and separation meant for pregnant patients admitted as inpatients. Our study demonstrates that the idea and practice of social isolation had specific significance and implications for pregnant patients, particularly when COVID testing was performed in preparation for admission to the hospital for delivery. For this population, isolation meant potential separation from family or other support persons during labor (e.g., doula). It also meant separation from the infant after birth, affecting the ability to see and hold the baby, skin-on-skin contact, breastfeeding, and bonding. While many healthcare systems did not have policies (or modified their policies) regarding obstetric patient isolation over the pandemic, stories of the experiences of other pregnant patients gained through personal experience or social media. Stories of those who had to labor without family present and who could not hold their newborn reverberated among the group. Studies demonstrate the needs of different type of support obstetric patients may when admitted for inpatient management. This includes patients who desire a support person during routine labor and delivery and those who experience an obstetric emergency that requires immediate intervention [31, 32]. These concerns must also be extended to patients who may experience preterm labor of a pre-viable newborn or undergoing induction of labor for a fetal loss. Data emerging from perinatal care during the pandemic support these concerns, particularly the impact of separation from family, support persons, and infants during the pandemic [33–36]. Our study findings highlight the priority to obtain data about the needs and concerns of pregnant patients who tested positive for COVID-19.

A second important factor contributing to testing hesitancy pertained to the ramifications of a positive test result on healthcare delivery, particularly during inpatient admissions for delivery. Study participants expressed several concerns about diminished quality of care and patient experience during the birthing process. Specifically, there was the issue of whether and to what extent healthcare providers would be delayed in preparing to provide routine or emergent care, given the amount of PPE needed and their familiarity with the donning process. They were also concerned about the chance of stigmatization by healthcare providers if they were positive, and this led to questions about the decisions and actions the pregnant individual had taken during pregnancy. Indeed, studies have demonstrated how the pandemic impacted the quality of maternal healthcare, including inpatient services [35, 36]. Studies also document the type and amount of stigmatization experienced by pregnant persons who tested positive for COVID [37–39]. This is concerning, given the degree to which the behaviors and choices of pregnant persons have been scrutinized before the pandemic [40]. Finally, it is important to highlight that there was uncertainty and trepidation about the available evidence to guide obstetric management if a patient were COVID-positive, particularly as there were delays with emerging data about how to prevent and manage SARS-CoV-2 in this medically complex population.

While there was significant pressure to implement testing during the pandemic in a time-sensitive fashion, these findings highlight the need for strategies that address the needs of different patient populations, particularly given the support for a universal testing approach for this population [41, 42]. For instance, participants recommended the establishment of separate testing facilities to test pregnant patients, enabling them to comply with testing recommendations from their healthcare provider with reduced concerns about viral exposure in the process and the myriad of concerns that come with a COVID positive result. Additionally, participants called for clear communication and transparency about hospital policies regarding the management of patients who are COVID-positive or decline testing. Our study findings also call for the need to include pregnant patients as key stakeholders in developing guidance about developing and implementing strategies for this unique patient population. Their voice, with respect to experiences during this pandemic and concerns for future pandemic management, has a vital role in the successful implementation of public health strategies. It is also essential to develop strategies that are cautious when data do not yet exist about the public health threat on pregnant patients without restricting the choices they can make because of a delay in the inclusion of pregnant persons in clinical studies. As stakeholders, pregnant patients can speak to how to find this balance while also attending to the priority of controlling an infectious threat throughout the population.

While our study provides insights into COVID test utilization and decline among pregnant patients, the findings should be contextualized with the limitations of this study. The study was based on patients from two healthcare systems in Ohio that adopted telehealth protocols similarly during the pandemic. Nonetheless, it is possible that there were subtle differences in the ways in which participants learned about and accessed COVID testing in addition to the clinical ramifications of the choice to accept or decline testing. In addition, participants, most of whom accepted COVID testing, self-selected for study participation. While these participants could speak to reasons why pregnant patients may decline testing, the perspectives of those individuals were not broadly represented in this population. Although we sought a broad demographic representation in our recruitment efforts, most participants were < 35 years of age, self-described race as White, and had at least one prior pregnancy. Different patient characteristics, including experience with the healthcare systems before, during, and after pregnancy, may also affect perspectives. We acknowledge that other healthcare systems and geographic areas of the U.S. may have had other experiences or practices with respect to COVID-19 testing education, delivery, and management. While our sample represented patients of different reproductive histories, our sample was limited in racial and ethnic representation. Despite these limitations, the study brings to light important findings for which further research is needed to elucidate pregnant patients' access and use of COVID testing among larger and more diverse patient populations and at different stages of the pandemic.

## Conclusion

A significant public health emergency, such as the COVID-19 pandemic, requires rapid and effective action with respect to infection prevention and management. While mechanisms such as COVID testing are necessary steps to mitigate viral spread across all populations, testing strategies must be individualized for special populations. This need for such an approach was apparent among pregnant patients, a population for whom it was essential to prevent infection, given the known medical and obstetric complications associated with infection. At the same time, this is a population who had unique concerns regarding the stigmatization of a positive COVID test result, the risks and benefits associated with testing procedures, and the impact of the choice to undergo or decline testing on their pregnancy and experiences during antepartum, intrapartum, and postpartum care. This is also a population for which it is critical to preserve autonomy and pregnant patients' ability to make voluntary informed decisions about their healthcare that reflect their needs and priorities without bias, stigmatization, or coercion from others. The findings of this study speak to the need for targeted strategies and education for this population to ensure that they receive evidence-based and patient-centered care during their pregnancy, even when public health strategies are implemented. They also raise important questions about weighing public versus individual health interests in the setting of an infectious pandemic and how to do so in a way that addresses the unique aspects of pregnancy and the needs of pregnant patients.

## Declarations

**Ethics approval and consent to participate:** This study has complied with all relevant national regulations and institutional policies of the Cleveland Clinic. It has been approved by the authors' Institutional Review Board. The study has been conducted in accordance with the Declaration of Helsinki (as revised in 2013). Informed consent was obtained from all individuals included in this study or their legal guardians or wards.

**Consent for publication:** Not Applicable.

**Availability of data and materials:** Additional data from interviews can be found in Table 2. Other data can be obtained on request from the corresponding author.

**Competing interests:** The author(s) declared no potential conflict of interest with respect to the research, authorship, and publication of this article.

**Funding:** The author(s) disclosed receipt of the following financial support for the research, authorship, and/or publication of this article: This work was supported by the National Center for Advancing Translational Sciences (UL1TR002548).

**Authors' contributions:** R.F. was responsible for the conception, design, analysis, interpretation, drafting, and final version of the work. C.D. was responsible for the acquisition of data, analysis, drafting, and final version of work; R.P. was responsible for the conception design, interpretation, and final version of the work; E.D. was responsible for the conception design, understanding, and final version of work, C.C. was responsible for acquisition of data, drafting and final version of work.

**Acknowledgments:** We'd like to acknowledge Kassandra Spates-Harden, Mary Jo Allen, Wendy Spencer, and Dr. Kelly Gibson for their contributions to the study development and recruitment.
